# Assessing gross motor function in cerebral palsy: What standardized scores do *not* tell us

**DOI:** 10.1111/dmcn.16480

**Published:** 2025-08-19

**Authors:** Peter Rosenbaum

**Affiliations:** ^1^ McMaster University – Paediatrics, CanChild Hamilton Ontario Canada

## Abstract

**This commentary is on the original article by Duran et al. on pages** 218–226 **of this issue.**

Developed in the late 1980s, the Gross Motor Function Measure (GMFM)^1^ was among the first formally validated clinical tools to evaluate change over time in gross motor function in young people with cerebral palsy (CP). Its current interval‐level version has 66 items and is widely used around the world. Duran et al.^2^ offer an elegant, innovative but complex statistical approach to the analysis of GMFM‐66 change scores using extensive longitudinal data available in their Centre for Prevention and Rehabilitation at the University of Cologne: ‘The centile curves … agreed very well with the reference curves of Hanna et al. and Smits et al. It therefore seems clearer in everyday clinical practice to use only one GMFM‐66 centile curve.’ The main advantage of their findings appears to be that these data expand the age range of previous studies.

Reading this paper elicited several reflections. The first is delight that the GMFM (with which our research group [*CanChild* Centre] is closely connected) continues to be both used and further explored, as has happened in this study. At the same time, as a clinician and health services researcher, I find both the methods and the potential application of the approach that has been validated here to be complex and likely beyond the scope of many frontline clinicians.

A second observation is that the extent and relative intensity of therapy services for young people with CP reported here is considerably greater than are available in most places. Thus, if in fact physiotherapy is making a major difference to the functional status of these children and adolescents, the findings from this study may have limited generalizability across the world, where therapy resources are much more limited.

This leads to my third and, I believe, most important thought. When the GMFM was created over 35 years ago, clinicians and health services researchers alike implicitly believed that whatever we were assessing, we needed an account of *capacity* (i.e. best performance under standard conditions). Our interventions were directed at what the World Health Organization's International Classification of Functioning, Disability and Health (ICF) framework^3^ would now call body structure and function. We made an assumed connection between improvements in those aspects of function (as assessed by changes in scores on standardized measures like GMFM) and overall functioning in daily life. Such assumptions and associations were rarely if ever assessed and reported.

In the past two decades, spurred in part by the influence of the ICF, our thinking in the field of childhood disability has expanded, and shifted, to focus on young people's *everyday performance* of activities important to them in their own environments, with less attention to the quality of that functioning or to changes on scores in standardized measures.^4^ The imaginative work of Chagas et al. has led to the creation of the briefer Gross Motor Family Report (GM‐FR).^5^ This validated parent‐report measure of everyday gross motor performance in children's environments is built on, and correlates well with, the GMFM‐66. What is novel here is that we now are able to capture changes in functioning that matter to children, and to take advantage of the expertise and experience of parents to report these aspects of functioning efficiently.

These comments in no way invalidate the work of Duran et al. They do however lead me to wonder whether the particular value of these reference centiles might best be realized in controlled research studies designed to assess the impact of specific interventions for young people with CP. Of course, in such studies, the additional use of the GM‐FR would provide important complementary understanding of the relationship between formal GMFM‐66 assessments and changes in the everyday function of the young people with whom we work.

## DISCLOSURE

PR is a co‐developer of the several versions of the GMFM, the GMFCS, and the GM‐FR, and believes that this experience provides a perspective on the issues addressed in this commentary. It must also be noted that the GM‐FR is soon to be available for sale on the *CanChild* website. PR will receive no proceeds of these sales.

## Data Availability

Not required.
